# Balancing the View of C1q in Transplantation: Consideration of the Beneficial and Detrimental Aspects

**DOI:** 10.3389/fimmu.2022.873479

**Published:** 2022-03-24

**Authors:** Raneem Khedraki, Hirotsugu Noguchi, William M. Baldwin

**Affiliations:** ^1^ Department of Inflammation and Immunity, Lerner Research Institute, Cleveland Clinic, Cleveland, OH, United States; ^2^ Department of Biological, Geological, and Environmental Sciences, Cleveland State University, Cleveland, OH, United States

**Keywords:** complement, C1q, tissue resident macrophages, pattern recognition receptor, B cells, donor specific antibodies, antibody-mediated rejection

## Introduction

Interest in antibody-mediated injury to transplants was renewed with discovery that deposition of the complement split product C4d on capillaries of renal transplants was a marker of poor outcomes (1, 2). Subsequent adoption of C4d and circulating donor specific antibodies (DSA) as two criteria in the Banff classification for antibody-mediated rejection (AMR) strengthened the concept that complement was a major contributor to graft injury (3). Since then, several clinical tests have been introduced to assess complement activation by DSA (4–6). Similarly, therapeutic interventions targeting various components of the complement cascade have been evaluated for preventing ischemia reperfusion injury or treating antibody-mediated rejection (7). These approaches are based on voluminous evidence for the inflammatory effects of complement activation starting in 1895 with Jules Bordet’s discovery that serum components we now know as complement could cause lysis of bacteria (8). However, in the last 30 years evidence has accumulated that some complement components can prevent escalation of complement activation to inflammation. A prime example is C1q, a subcomponent of C1, the first component of the classical pathway of complement. Many clinical reports link deficiencies in C1q with increased inflammatory responses, autoantibodies and severe autoimmune disease resembling lupus (9, 10). Furthermore, experimental deletion of C1q in mice resulted in autoimmune phenotypes with increased autoantibody titers (11). Rejection of cardiac allografts was also accelerated in C1q deficient mice compared to normal controls (12). The accelerated rejection was associated with increased antibody titers in the circulation, IgG deposition in capillaries and neutrophil infiltrates in the cardiac allografts.

## C1q Functions as Part of the C1 Complex and Independently as a Pattern Recognition Receptor

C1 is a complex of 3 subcomponents: C1q, C1r and C1s. When Lepow and colleagues first isolated these 3 subcomponents, they reported that all 3 were required for initiation of the classical cascade (13). C1q was found to bind to antibody-antigen complexes, whereas C1r and C1s provided the protease activity to cleave subsequent complement components. In this context, C1q binds to the Fc portion of antibody. C1q has 6 globular heads to engage antibodies and optimal binding to IgG occurs when 6 IgG antibodies are arrayed in a hexamer formation (14). C1r and C1s stabilize C1q binding to IgG in addition to cleaving the downstream complement components C4 and C2 (15).

However, C1q has critical functions independent of antibodies or C1r and C1s. The independent functions of C1q predate both antibodies or C1r and C1s in evolutionary time (16). In lampreys, a primitive vertebrate that lacks immunoglobulin, the orthologue of mammalian C1q binds N-acetylglucosamine (17). In mammals, C1q retains pattern recognition receptor (PRR) functions (18). Isolated C1q binds directly to apoptotic cells when phosphatidylserine, calreticulin, DNA and other molecules are exteriorized (19–21). This PRR function of C1q facilitates ingestion of apoptotic bodies by macrophages (22). Systemic C1q causes efficient clearance of apoptotic bodies in the spleen by marginal zone macrophages (23). Importantly, C1q opsonized apoptotic bodies polarize macrophages towards non-inflammatory profiles (24). More recent studies have demonstrated that C1q also inhibits maturation of monocytes into dendritic cells and metabolically regulates T cells (25).

The disparate evolutionary origins and functions of C1q and the two C1 proteases are further underscored by the fact that these molecules are encoded by genes on different chromosomes (26, 27). The primary sources of C1q and the two C1 proteases are also different (**Table 1**). The majority of C1r and C1s is produced by hepatocytes (33), whereas C1q is primarily produced by macrophages and dendritic cells (34). Although macrophages can produce some C1r and C1s as well as C1q, early immunohistological studies suggested that C1q, C1r and C1s are frequently produced by different macrophages (35). This early observation is provocative in the context of new data about macrophage heterogeneity.

**Table 1:** Comparison of sources and functions of C1 related components.

C1 component	Chromosome (human)	Primary sources	Other Sources	Stimulation	Function
C1q	1p34.-1p36.3 (27)	Tissue resident macrophages > Infiltrating macrophages; Immature dendritic cells (27–31)	Epithelial cells, Endothelial cells (32)	IFNγ^1^ (27)	Pattern Recognition (13, 14); Binds antibodies (18–20)
C1r/C1s	12p13.31 (26, 33)	Liver (33)	Epithelial cells, Macrophages (34–36)	IFNγ (36)	Serine proteases that cleave C4 and C2; Stabilize C1q binding to antibodies (15)
C1 inhibitor	11q11-q13.1 (37)	Liver (37)	Monocytes, Macrophages, Endothelial Cells (34)	IFN types I and II, IL-6, IL-1, and TNFα (34, 37)	Serine protease inhibitor (serpin) (38)

^1^Modulation of C1q expression has been tested in peritoneal macrophages and cultured human macrophages, but not in tissue resident macrophages.

## Resident Macrophages as Sources of C1q

Techniques for cell lineage tracking have distinguished macrophages that infiltrate inflamed tissues from resident macrophages (28), and single cell RNA sequencing has provided transcriptional signatures for different macrophage populations in quiescent and inflamed organs (28–30). Various functions have been proposed for tissue resident macrophages, among which is clearance of tissue debris (30, 39). In this context it is notable that C1q has been identified as a marker of resident macrophages in kidney, heart and lung (28–30, 40, 41). Less data has been reported for C1r and C1s expression, but Pinto, et al. found in contrast to C1q transcripts, C1r and C1s transcripts were expressed at low levels in resident macrophages of hearts (30). In the quiescent kidney, C1r is primarily expressed by epithelial cells of distal tubules rather than macrophages, and activation of complement and recruitment of macrophages to inflamed kidneys is decreased greatly in C1r knockout mice (36).

## Effects of Transplantation on Resident Macrophages and C1q

Although upregulation of C1 expression has been reported in renal allo- and isografts in rats within hours after transplantation (42) as well as in biopsies from human renal transplants after perfusion is reestablished (43), the source of C1 and the kinetics of different subcomponents has not been established completely. In biopsies from a few renal transplants, Malone et al. (29) leveraged single nucleotide variation to demonstrate resident macrophages persisted up to 28 days after transplantation in non-rejecting human kidneys and these macrophages expressed high levels of C1q transcripts. In contrast, biopsies from grafts undergoing rejection contained only infiltrating macrophages that expressed low levels of C1q and high levels of CD68 and Fcγreceptor 3a. Similar findings have been reported for experimental heart allografts in mice (31). In this model, numbers of donor macrophages decreased within 14 days in allografts to mice without immunosuppression, but a subpopulation of donor macrophages, which was identified as CCR2-, persisted undiminished in recipients treated with immunosuppression. In contrast CCR2+ macrophages decreased regardless of immunosuppression. Donor CCR2- macrophages expressed higher levels of C1q than CCR2+ macrophages and only CCR2- macrophages were found to be essential for graft survival. Expression of C1r or C1s by these subpopulations of resident macrophages was not reported. Moreover, it has not been established whether C1q expression is modulated in resident macrophages after transplantation, although IFNγ has been demonstrated to stimulate C1q promoter activity in cultured macrophages derived from human blood (27). Similarly, IFNγ, IL-10 and dexamethasone increase expression of C1q by human monocytes isolated from blood (44), but the effects of these mediators on production of C1q and C1r or C1s by resident macrophages in tissues is not known.

Production of C1r by tubular epithelial cells is notable because expression of C1r and C1s transcipts have been reported to increase in human renal transplants during antibody or cell-mediated rejection (45, 46); particularly in conjunction with tubulitis (46). In a mouse model, C1q expression increases in proximal tubular epithelium during rejection (32). These data suggest that C1q is produced by subsets of resident macrophages while they persist in the graft, whereas both C1q and the C1 proteases are produced by tubular epithelial cells stimulated by IFNγ.

## C1 Inhibitor Eliminates C1r and C1s but Preserves C1q Function

Another component that determines the balance of C1q functions is C1 inhibitor (C1inh). C1inh regulates complement activation by covalently binding C1r and C1s and removing these proteases from C1q. By removing C1r and C1s from the C1 complex, structural studies indicate that C1inh increases the flexibility of C1q and enhances the PRR function of C1q (18, 38). C1inh is primarily produced in the liver, but the production of C1inh by other cells including macrophages and endothelial cells can be increased by IFNγ (37). Little is known about the production of C1inh by resident or infiltrating macrophages following transplantation. However, transcripts for C1inh have been reported to increase during chronic antibody-mediated rejection (47).

## Connecting C1q, Damaged Cells, Autoantibodies and Alloantibodies in Transplants

Most transplanted organs are retrieved from brain dead or non-heart beating donors and subjected to various periods of warm and cold ischemia. All these conditions increase injury to parenchymal cells and may alter resident macrophage functions (48). Experimental models have linked ischemic injury to increased inflammatory infiltrates and release of extracellular vesicles (49, 50). Although the immunogenicity of different subtypes of extracellular vesicles has not been fully established, proteomic assays have demonstrated some extracellular vesicles contain autoantigens and alloantigens (49, 51). At least some types of extracellular vesicles can deliver their antigenic content to antigen presenting cells (52). C1q could be a critical variable in determining the immune response to the antigenic content of extracellular vesicles that express phosphatidylserine and other potential ligands for C1q. In particular, B cell responses may be modulated by C1q as evidenced by increased auto- and alloantibody production in C1q deficient mice and humans (9–12). Even though antibodies to MHC antigens are more conclusively linked to graft injury and poor outcomes, there is increasing evidence that autoantibodies can contribute to graft damage (53). The range of autoantibodies implicated in graft injury include angiotensin II type I receptor, endothelin A, k-alpha tubulin, collagen V, vimentin and perlecan. Recently, antibody-mediated rejection has been associated with increases in antibodies associated with autoimmune diseases such as lupus, including IgG anti-Ro/Sjögren syndrome-antigen A (SS-A) and anti-major centromere autoantigen (CENP)-B (54).

## Discussion

Based on current knowledge, it is plausible that C1q production by resident macrophages promotes non-inflammatory clearance of injured tissue from transplanted organs. In contrast, the complete C1 complex initiates the classical cascade of complement. This interpretation would support strategies to preserve or enhance C1q production while inhibiting the activity of C1r and C1s. Two such therapeutic interventions have been tested in transplant recipients. One is C1inh (55, 56) and the other is a monoclonal antibody to C1s (57, 58). Both biologics have been tested in patients experiencing antibody mediated rejection (**Table 2**). In general, this approach has decreased complement activation temporarily, but has little or no effect on long-term outcomes (58–60). Conversely, several experimental studies indicate that C1inh can be effective in decreasing injury due to ischemia-reperfusion (61–64). One extended clinical study included 70 recipients of deceased donor kidney transplants at risk for delayed graft function. C1inh (n=35) versus placebo (n=35) administered intraoperatively and at 24 hours resulted in decreased cumulative incidence of graft failure and higher eGFR over 3.5 years (55, 56). These clinical studies have been designed to prevent initiation of the complement cascade by C1, and the effects on the PRR function of C1q were not examined. With increasing application of *ex vivo* perfusion of organs before transplantation, enhancing or preserving C1q PRR functions should be considered. This could be accomplished by eliminating CCR2^+^ C1q^lo^ resident macrophages, while preserving CCR2^-^C1q^hi^ resident macrophages (31). Alternatively or additionally, biologically modified C1q could be added to the perfusate (65). Various mutant C1q molecules have been described that retain PRR function but lack binding sites for C1r or C1s (66). C1q is 460 kDa and can diffuse across endothelial barriers into the interstitial spaces. As a result, perfusates containing C1q would supplement C1q in the interstitium of organs. We propose that increasing C1q function locally would increase local and systemic clearance of apoptotic and necrotic cells from transplants and modulate sensitization (23, 30, 39). While abundant evidence indicates that C1q modulates the immune responses to autoantigens, little data is available regarding alloimmune responses. The one study of cardiac allografts in C1q deficient mice only investigated the effects of C1q deficiency in the recipient (12). Although absence of C1q in the recipient resulted in increased donor specific alloantibody titers, the recent data demonstrating resident macrophages are potent sources of C1q suggests that testing the effects of C1q deficiency in the donor would be informative especially in models where warm and cold ischemic times are extended to reflect the ischemic times incurred by clinical organ transplants retrieved from deceased donors (50, 67, 68).

**Table 2:** Clinical Trials of C1inh and anti-C1s treatment.

Treatment Protocol	Cohort	Primary Outcome	Secondary Outcome	Ref
Anti-C1s mAb: 4 weekly doses (60 mg/kg)	Stable kidney transplant recipients with late active ABMR (n=10)	5 of 8 recipients with C4d-positive biopsies became C4d-negative in 5-week follow-up	No change in microcirculation inflammation, gene expression patterns, DSA levels, or kidney function	(58)
C1inh: 20000 units divided in 7 doses on alternate days added to conventional IVIg and plasmapheresis	Biopsy-proved AMR with concurrent DSAs (n= 9 placebo; 9 C1inh)	No difference in day 20 pathology or graft survival	Six-month biopsies (n=14): Transplant glomerulopathy in 0 of 7 C1 INH treated; 3 of 7 controls	(59)
C1inh: 20 units/kg for 3 days, then twice weekly added to high dose IVIg for 6 months	Kidney recipients with non-responsive active ABMR (n=6)	Improved eGFR at 6 months after inclusion	No change in histological features, except a decrease in the C4d deposition	(60)
C1inh: 50 units/kg intraoperatively and at 24 hours	Deceased donor kidney transplant recipients at risk for delayed graft function (n=35 placebo; n=35 C1inh)	Decreased the cumulative incidence of graft failure over 3.5 years	Higher eGFR over 3.5 years	(55)

In summary, while the function of C1q as part of the C1 complex that initiates the classical complement cascade has been extensively examined in the transplant field especially in the context of antibody-mediated rejection, greater appreciation of the anti-inflammatory functions of C1q could open novel approaches to limiting graft injury.

## Author Contributions

All authors participated in reviewing the literature, writing and editing the manuscript. RK and HN performed experiments that led to the literature search and hypothesis. All authors approved the submitted version.

## Funding

All authors are supported by grants NIH R01 AI165513 and PO1 AI087586 from the NIAID.

## Conflict of Interest

The authors declare that the research was conducted in the absence of any commercial or financial relationships that could be construed as a potential conflict of interest.

## Publisher’s Note

All claims expressed in this article are solely those of the authors and do not necessarily represent those of their affiliated organizations, or those of the publisher, the editors and the reviewers. Any product that may be evaluated in this article, or claim that may be made by its manufacturer, is not guaranteed or endorsed by the publisher.
